# High comprehensive complication index after minimally invasive esophagectomy associated with poor short-term and long-term outcome: a propensity score matching analysis

**DOI:** 10.3389/fonc.2025.1661797

**Published:** 2025-10-28

**Authors:** Xiaoqing Wang, Jingchuan Yu, Shuhan Xie, Ye Lin, Peipei Zhang, Lei Gao, Zhinuan Hong, Mingqiang Kang

**Affiliations:** ^1^ Department of Thoracic Surgery, Fujian Medical University Union Hospital, Fuzhou, China; ^2^ Key Laboratory of Ministry of Education for Gastrointestinal Cancer, Fujian Medical University, Fuzhou, China; ^3^ Fujian Key Laboratory of Tumor Microbiology, Fujian Medical University, Fuzhou, China; ^4^ Clinical Research Center for Thoracic Tumors of Fujian Province, Fuzhou, China; ^5^ Key Laboratory of Cardio-Thoracic Surgery, Fujian Medical University, Fuzhou, China

**Keywords:** overall survival, comprehensive complication index, Clavein-Dindo classification, minimally invasive esophagectomy, esophageal squamous cell carcinoma

## Abstract

**Introduction:**

The comprehensive complication index (CCI) is a valuable index to comprehensively and systematically evaluate complication severity. This study aimed to evaluate the predictive ability of comprehensive complication index on short- and long-term overall survival(OS) in patients with esophageal squamous cell carcinoma (ESCC) undergoing Mckeown minimally invasive esophagectomy (MIE).

**Methods:**

A total of 320 patients treated with radical MIE from 2013 to 2017 were included, and the primary outcome was OS. Firstly, the optimal cut-off value of CCI was determined by X-tile. Propensity score matching(PSM) was used to balance the baseline characteristics. Second, postoperative hospital stay and hospital costs between high- and low-CCI groups were compared. Third, the Kaplan-Meier survival curve was used to analyze survival differences. Fourth, Cox analysis was used to explore the risk factors of OS. Fifth, univariate and multivariate logistic analysis was used to determine the risk factors of high CCI.

**Results:**

The patients with CCI > 24.2 was defined as high-CCI group, and those with CCI ≤ 24.2 were assigned to low-CCI group. The high-CCI group had more hospital costs and longer hospital stays than the low-CCI group before and after PSM (both p<0.001). The Kaplan-Meier survival curve indicated that high-CCI group had worse prognosis both before and after PSM (before matching: P<0.001; after matching: P = 0.01). CCI was determined as an independent prognostic factor (before PSM, P = 0.001; after PSM, P = 0.003).

**Conclusion:**

The CCI could quantify postoperative complications after esophagectomy. High CCI was associated with longer postoperative hospital stays and expenses and is an independent risk factor for poor OS, holding great vlaue for reference for medical insurance, surgical quality and prognosis management.

## 1 Introduction

Esophageal cancer (EC) is one of the most common and challenging types of cancer, with 572,000 new cases diagnosed each year and 500,000 deaths (1). At present, due to population aging, EC mainly occurs in middle-aged and elderly people, with the average age of diagnosis being 67 years old, and about 30% of patients are over 75 years old (2, 3). For patients with EC, surgical resection is the main treatment. Although minimally invasive esophagectomy (MIE) has become a popular surgical method in recent years to reduce postoperative complications, the incidence of postoperative complications is still relatively high (4).

Postoperative complications were often considered practical indicators to evaluate surgical quality, surgical safety. Review the history of medical classification of complications, from the initial simple classification of complications into major complications and minor complications (5). Then, the Clavien-Dindo complication classification is created, and complication classification is used to evaluate the postoperative complications (6). However, surgeons often only focus on severe complications and ignore other minor complications. The lack of a comprehensive assessment of all postoperative complications in the Clavien-Dindo complication has led clinicians to propose a CCI that provides a comprehensive picture of the true overall complication profile. The CCI has been confirmed as a comprehensive prognostic factor for the short-term and long-term outcomes in patients with gastric cancer (7). The role of CCI in patients undergoing MIE for ESCC was still unclear.

Compared with open esophagectomy, MIE could accelerate the perioperative recovery of patients without affecting the long-term prognosis in patients with EC (8, 9). Postoperative complications after MIE (such as anastomotic leakage) could greatly affect the prognosis of EC (10, 11). Perioperative medical quality (especially the avoidance and management of complications) also has a profound impact on the long-term survival of patients with esophageal cancer (12). However, the impact of one specified complication for postoperative complications was still controversial. This difference was partly due to the fact that Clavien-Dindo complication grading could only provide a qualitative evaluation rather than a quantitative evaluation of postoperative complications. In this study, we aimed to investigate the predictive ability of the CCI for short-term prognosis (postoperative hospital stay and surgical cost) and long-term prognosis (overall survival, OS) in patients undergoing MIE for esophageal squamous cell carcinoma (ESCC).

## 2 Methods

### 2.1 Patient selection

**Figure 1:**
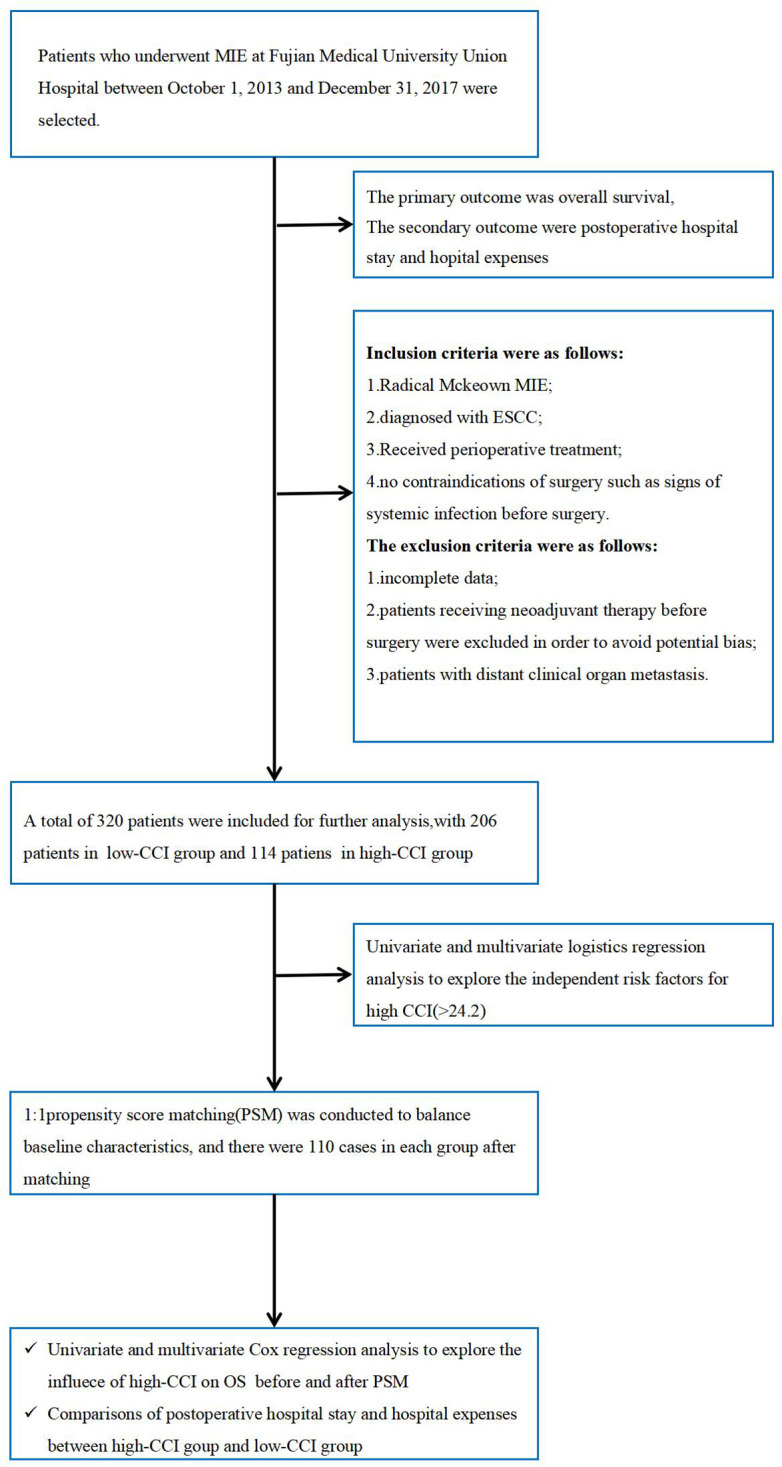
Patient selection and statistical analysis flow chart.

Patients who underwent MIE at Fujian Medical University between October 1, 2013 and December 31, 2017 were selected. This study was approved by the Institutional Review Board in Fujian Medical University Union Hospital(IRB number 2022YK202). Inclusion criteria were as follows: 1. Radical Mckeown MIE; 2. Diagnosed with ESCC; 3. Received perioperative treatment(including preoperative examination, operation, and postoperative nursing); 4. No contraindications of surgery. The exclusion criteria were as follows: 1. incomplete clinical data; 2. Patients who received neoadjuvant therapy were excluded due to the small sample size before 2017 and potential survival differences among regimens, to avoid bias; 3.Patients diagnosed with cM1.The details of the patient selection and analysis flowchart are presented in **Figure 1**.

### 2.2 Data collection and outcome definition

In this study, the primary outcome was overall survival (OS), which was defined as the time from surgery to death or the last follow-up. The secondary outcomes were postoperative hospital stay and hospital costs. Postoperative complications Clavien-Dindo ≥ 3a were defined as major complications, and postoperative complications Clavien-Dindo < 3a was defined as minor complication (13). Naples score (NPS) was calculated based on the dichotomous variables of neutrophil/lymphocyte ratio, lymphocyte/monocyte ratio, serum albumin, and total cholesterol (14). NPS = 0 was defined as a low Naples score, NPS = 1 or 2 was defined as a medium Naples score, and NPS = 3 or 4 was defined as a high Naples score (15). Preoperative comorbidities were graded using the Charson comorbidities Index. Patients with a Charson index of less than or equal to 1 point were considered a low-risk group, while patients with a Charson index of more than 1 point were considered a high-risk group (16, 17).

### 2.3 Treatment protocol

All patients underwent MIE, including gastroesophageal replacement for digestive tract reconstruction and left cervical anastomosis (McKeown operation, 18, 19). Thoracic and abdominal lymph nodes were routinely dissected, and three-field lymph node dissection was performed for patients with suspected cervical lymph node metastasis. During the periof of 2013 to 2017, patients diagnosed with cT1–2 or cN_0_ were recommended to receive surgery first. Some patients with cT3 or cN+ underwent surgery first, based on surgical feasibility assessed by the surgeon and the patient’s treatment preference.

Patients were followed up every 3 to 6 months in the first and second years and every six months in the third year. The patients were followed up once a year from the fifth year after surgery.

### 2.4 Calculation of CCI

The CCI was calculated based on the Clavien-Dindo grading system to reflect the comprehensive severity of postoperative complications. The formula was the sum of ownership weights divided by the square root of 2 equals the CCI. The final detailed formula for the CCI was: CCI = √ (median reference value from physicians(MRVphys) × median reference value from patients(MRVpat)/2. The value of CCI was continuous, ranging from 0 (no complications) to 100 (death). We used the online calculator (http://www.assessurgery.com) to calculate CCI (20).

### 2.5 Statistical analysis

Categorical variables were expressed as percentages and compared with the Chi-square test/Fisher exact test. Continuous variables were represented by medians and compared by the Mann-Whitney U test. In this study, X-tile software was used to calculate the optimal cut-off value of the CCI (21, 22). The operative duration was divided into the long-and short- duration group using the receiver operating characteristic curve. First, PSM was used to match the variables with differences between the two groups. The caliper value was set as 0.02, and the matching ratio was 1:1. The paramaters includes sex, age, BMI, Charson comorbidity index, tumor location, smoking history, CEA, vascular tumor thrombus, T stage, N stage, Grade, NPS and adjuvant therapy. Secondly, the postoperative hospital stay and postoperative hospitalization cost between the two groups were compared. Third, independent risk factors were determined by univariate and multivariate Cox regression analysis (23). Statistical analysis was performed using X-tile 3.6.1 software (Yale University, USA) and R software version 4.0.5 (http://www.r-project.org). All statistical test levels considered a P value less than 0.05 to be statistically significant.

## 3 Results

### 3.1 Patient characteristics

A total of 320 patients were included in this study. 258 (80.62%) patients were ≤65 years old, and 240 (75%) were males. All patients underwent McKeown MIE, and 302 patients (94.38%) underwent 2-field lymph node dissection. The detail information of ESCC patients was shown in **Supplementary Table S1**.

### 3.2 Comparison of high-CCI group and low-CCI group before and after PSM

The X-tile software determined an optimal CCI cutoff of 24.2, dividing patients into high- and low-CCI groups. Before PSM, there were significant differences in T stage between the groups. After PSM, the baseline characteristics were balanced, as is shown in **Table 1**.

**Table 1:** The baseline characteristics between high CCI group and low CCI group before and after PSM.

Contents	Before PSM			After PSM		
	Low CCI group	High CCI group	P	Low CCI group	High CCI group	P
Sex			0.225			1.000
male	150 (72.82%)	90 (78.95%)		87 (79.09%)	87 (79.09%)	
female	56 (27.18%)	24 (21.05%)		23 (20.91%)	23 (20.91%)	
Age			0.147			0.873
≤65	171 (83.01%)	87 (76.32%)		84 (76.36%)	85 (77.27%)	
>65	35 (16.99%)	27 (23.68%)		26 (23.64%)	25 (22.73%)	
BMI			0.182			0.964
≤ 18.5	24 (11.65%)	7 (6.14%)		8 (7.27%)	7 (6.36%)	
18.5–25	147 (71.36%)	91 (79.82%)		86 (78.18%)	87 (79.09%)	
>25	35 (16.99%)	16 (14.04%)		16 (14.55%)	16 (14.55%)	
Charson comorbidity index			0.94			1.000
0-1	192 (93.20%)	106 (92.98%)		102 (92.73%)	102 (92.73%)	
≥2	14 (6.80%)	8 (7.02%)		8 (7.27%)	8 (7.27%)	
Tumor location			0.751			0.881
upper	17 (8.25%)	12 (10.53%)		13 (11.82%)	11 (10.00%)	
middle	146 (70.87%)	77 (67.54%)		75 (68.18%)	75 (68.18%)	
lower	43 (20.87%)	25 (21.93%)		22 (20.00%)	24 (21.82%)	
Smoking history			0.131			0.779
no	92 (44.66%)	41 (35.96%)		39 (35.45%)	41 (37.27%)	
yes	114 (55.34%)	73 (64.04%)		71 (64.55%)	69 (62.73%)	
CEA			0.354			1.000
normal	189 (91.75%)	101 (88.60%)		98 (89.09%)	98 (89.09%)	
abnormal	17 (8.25%)	13 (11.40%)		12 (10.91%)	12 (10.91%)	
Vascular tumor thrombus			0.052			0.762
negative	166 (80.58%)	81 (71.05%)		79 (71.82%)	81 (73.64%)	
positive	40 (19.42%)	33 (28.95%)		31 (28.18%)	29 (26.36%)	
T stage			<0.001			0.697
T1	78 (37.86%)	26 (22.81%)		30 (27.27%)	26 (23.64%)	
T2	37 (17.96%)	12 (10.53%)		9 (8.18%)	12 (10.91%)	
T3	91 (44.17%)	76 (66.67%)		71 (64.55%)	72 (65.45%)	
N stage			0.245			0.604
N0	123 (59.71%)	55 (48.25%)		63 (57.27%)	54 (49.09%)	
N1	43 (20.87%)	33 (28.95%)		23 (20.91%)	30 (27.27%)	
N2	34 (16.50%)	22 (19.30%)		21 (19.09%)	22 (20.00%)	
N3	6 (2.91%)	4 (3.51%)		3 (2.73%)	4 (3.64%)	
Grade			0.427			0.96
G1	72 (34.95%)	47 (41.23%)		46 (41.82%)	44 (40.00%)	
G2	115 (55.83%)	55 (48.25%)		52 (47.27%)	54 (49.09%)	
G3	19 (9.22%)	12 (10.53%)		12 (10.91%)	12 (10.91%)	
NPS score			0.480			0.782
0	61 (29.61%)	28 (24.56%)		32 (29.09%)	28 (25.45%)	
1-2	114 (55.34%)	71 (62.28%)		63 (57.27%)	68 (61.82%)	
3-4	31 (15.05%)	15 (13.16%)		15 (13.64%)	14 (12.73%)	
Adjuvant therapy			0.906			0.893
no	99 (48.06%)	54 (47.37%)		52 (47.27%)	51 (46.36%)	
yes	107 (51.94%)	60 (52.63%)		58 (52.73%)	59 (53.64%)	

The high CCI group had more medical expenses than the low CCI group (before PSM: 97098yuan vs. 84832yuan, P<0.001; after PSM: 99342yuan vs. 84590yuan, P<0.001) and longer hospital stays after surgery (before PSM:16 days vs. 9 days, P<0.001; after PSM: 16 days vs. 9 days, P<0.001), as is shown in **Supplementary Table S2**.

Besides, the comparisons of detailed complications between the two groups before and after PSM were summarized in **Table 2**.

**Table 2:** The comparisons of detailed complications between high CCI group and low CCI group before and after PSM.

Contents	Before PSM			After PSM		
	Low CCI group	High CCI group	P	Low CCI group	High CCI group	P
Pneumonia			<0.001			<0.001
no	192 (93.20%)	26 (22.81%)		102 (92.73%)	26 (23.64%)	
yes	14 (6.80%)	88 (77.19%)		8 (7.27%)	84 (76.36%)	
Pleural Effusion			<0.001			<0.001
no	200 (97.09%)	81 (71.05%)		107 (97.27%)	78 (70.91%)	
yes	6 (2.91%)	33 (28.95%)		3 (2.73%)	32 (29.09%)	
Pneumothorax			0.001			0.072
no	204 (99.03%)	104 (91.23%)		109 (99.09%)	103 (93.64%)	
yes	2 (0.97%)	10 (8.77%)		1 (0.91%)	7 (6.36%)	
Intestinal bleeding or obstruction			0.241			0.366
no	204 (99.03%)	110 (96.49%)		109 (99.09%)	106 (96.36%)	
yes	2 (0.97%)	4 (3.51%)		1 (0.91%)	4 (3.64%)	
Arrhythmia			<0.001			0.002
no	203 (98.54%)	97 (85.09%)		107 (97.27%)	94 (85.45%)	
yes	3 (1.46%)	17 (14.91%)		3 (2.73%)	16 (14.55%)	
Anastomotic leakage			<0.001			<0.001
no	200 (97.09%)	65 (57.02%)		108 (98.18%)	63 (57.27%)	
yes	6 (2.91%)	49 (42.98%)		2 (1.82%)	47 (42.73%)	
Liver dysfunction			<0.001			<0.001
no	157 (76.21%)	58 (50.88%)		90 (81.82%)	57 (51.82%)	
yes	49 (23.79%)	56 (49.12%)		20 (18.18%)	53 (48.18%)	
Recurrent laryngeal nerve injury			0.313			0.614
no	199 (96.60%)	113 (99.12%)		107 (97.27%)	109 (99.09%)	
yes	7 (3.40%)	1 (0.88%)		3 (2.73%)	1 (0.91%)	
Surgical Site Infection			0.241			0.679
no	204 (99.03%)	110 (96.49%)		108 (98.18%)	106 (96.36%)	
yes	2 (0.97%)	4 (3.51%)		2 (1.82%)	4 (3.64%)	
Chylous Leakage			0.106			0.366
no	205 (99.51%)	110 (96.49%)		109 (99.09%)	106 (96.36%)	
yes	1 (0.49%)	4 (3.51%)		1 (0.91%)	4 (3.64%)	

### 3.3 Survival analysis of CCI before and after PSM

**Figure 2:**
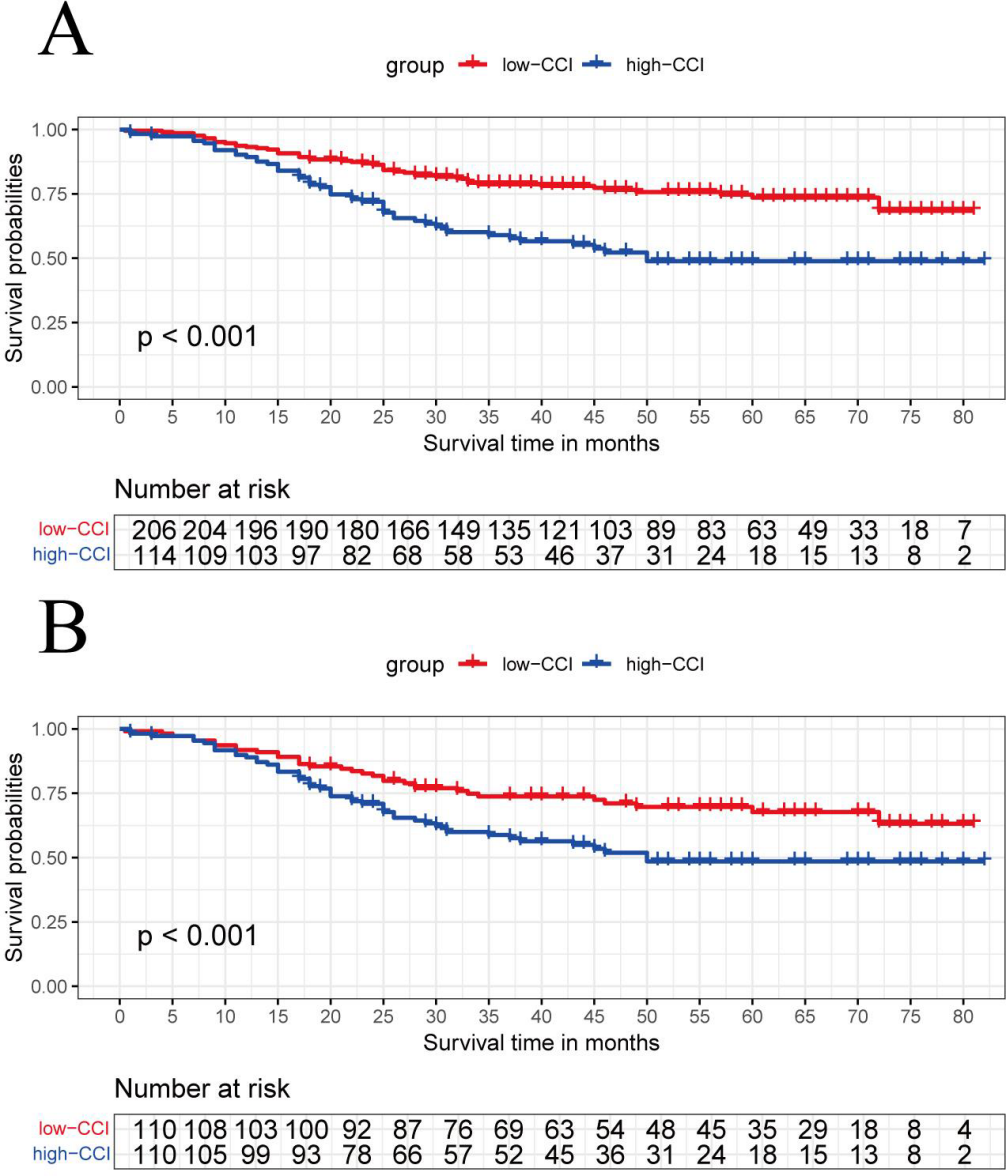
The Kaplan-Meier curve analysis compares the overall survival between the high-CCI group and the low-CCI group before **(A)** and after matching **(B)**.

Kaplan-Meier survival curves were analyzed for groups with high- and low- CCIs before and after PSM. Before matching, patients in the high-CCI group had worse survival outcomes than those in the low-CCI group (**Figure 2A**, P < 0.001). After matching, patients with the higher CCI still showed a worse prognosis (**Figure 2B**, P < 0.001).

Before PSM, univariate Cox analysis showed that the influencing factors of OS after esophageal cancer surgery were vascular thrombus, T stage, N stage, adjuvant therapy, CCI and NPS score. In the following multivariate Cox analysis, the independent influencing factors of OS were T stage, N stage, and CCI (P < 0.001). Similarlly, after PSM, univariate and multivariate Cox analysis showed the independent influencing factors were the T stage, N stage, and CCI (P = 0.015), as is shown in **Table 3**.

**Table 3:** The univariate and multivariate Cox analysis for OS before PSM and after PSM.

Contents	before PSM				after PSM			
	Univariate HR(95%CI)	P	Multivariate HR(95%CI)	P	Univariate HR(95%CI)	P	Multivariate HR(95%CI)	P
Sex								
male	Reference				Reference			
female	0.65 (0.39 ~ 1.06)	0.086			0.72 (0.40 ~ 1.27)	0.255		
Age								
≤65	Reference				Reference			
>65	1.43 (0.91 ~ 2.25)	0.123			1.04 (0.63 ~ 1.72)	0.887		
BMI								
≤ 18.5	Reference				Reference			
18.5–25	0.92 (0.47 ~ 1.78)	0.798			0.69 (0.32 ~ 1.52)	0.359		
>25	1.08 (0.50 ~ 2.35)	0.84			0.71 (0.28 ~ 1.81)	0.475		
Charson comorbidity index								
0-1	Reference				Reference			
≥2	0.80 (0.35 ~ 1.83)	0.596			0.73 (0.30 ~ 1.81)	0.496		
Tumor location								
upper	Reference				Reference			
middle	1.86 (0.81 ~ 4.28)	0.145			2.06 (0.89 ~ 4.77)	0.092		
lower	1.80 (0.73 ~ 4.45)	0.200			1.50 (0.58 ~ 3.87)	0.401		
Smoking history								
no	Reference				Reference			
yes	1.33 (0.88 ~ 1.99)	0.177			1.22 (0.77 ~ 1.94)	0.388		
CEA								
normal	Reference				Reference			
abnormal	0.92 (0.46 ~ 1.82)	0.809			0.95 (0.47 ~ 1.89)	0.875		
Vascular tumor thrombus								
negative	Reference				Reference			
positive	2.34 (1.56 ~ 3.51)	<0.001			2.02 (1.30 ~ 3.13)	0.002		
T stage								
T1	Reference		Reference		Reference		Reference	
T2	2.55 (1.20 ~ 5.43)	0.015	1.99 (0.92 ~ 4.29)	0.081	3.84 (1.43 ~ 10.32)	0.008	2.83 (1.04 ~ 7.68)	0.042
T3	4.79 (2.65 ~ 8.66)	<0.001	2.70 (1.44 ~ 5.06)	0.002	4.90 (2.25 ~ 10.68)	<0.001	2.70 (1.19 ~ 6.13)	0.017
N stage								
N0	Reference		Reference		Reference		Reference	
N1	2.56 (1.53 ~ 4.28)	<0.001	1.96 (1.16 ~ 3.32)	0.012	3.06 (1.70 ~ 5.50)	<0.001	2.47 (1.36 ~ 4.49)	0.003
N2	5.16 (3.15 ~ 8.44)	<0.001	3.70 (2.20 ~ 6.21)	<0.001	6.18 (3.55 ~ 10.78)	<0.001	4.81 (2.67 ~ 8.64)	<0.001
N3	8.51 (3.88 ~ 18.66)	<0.001	6.06 (2.71 ~ 13.53)	<0.001	10.64 (4.24 ~ 26.71)	<0.001	6.98 (2.70 ~ 18.02)	<0.001
Grade								
G1	Reference				Reference			
G2	1.13 (0.74 ~ 1.73)	0.572			1.27 (0.79 ~ 2.02)	0.322		
G3	1.00 (0.49 ~ 2.02)	0.998			1.03 (0.49 ~ 2.18)	0.93		
NPS score								
0	Reference				Reference			
1-2	1.73 (1.04 ~ 2.88)	0.036			1.68 (0.95 ~ 2.99)	0.076		
3-4	1.70 (0.88 ~ 3.31)	0.117			2.21 (1.07 ~ 4.58)	0.033		
Adjuvant therapy								
no	Reference				Reference			
yes	1.94 (1.28 ~ 2.94)	0.002			1.57 (1.01 ~ 2.46)	0.050		
CCI								
low-CCI	Reference		Reference		Reference		Reference	
high-CCI	2.24 (1.51 ~ 3.32)	<0.001	1.93 (1.29 ~ 2.88)	0.001	1.79 (1.15 ~ 2.78)	0.010	1.75 (1.12 ~ 2.73)	0.015

### 3.4 Subgroup analysis of CCI before and after PSM

**Figure 3:**
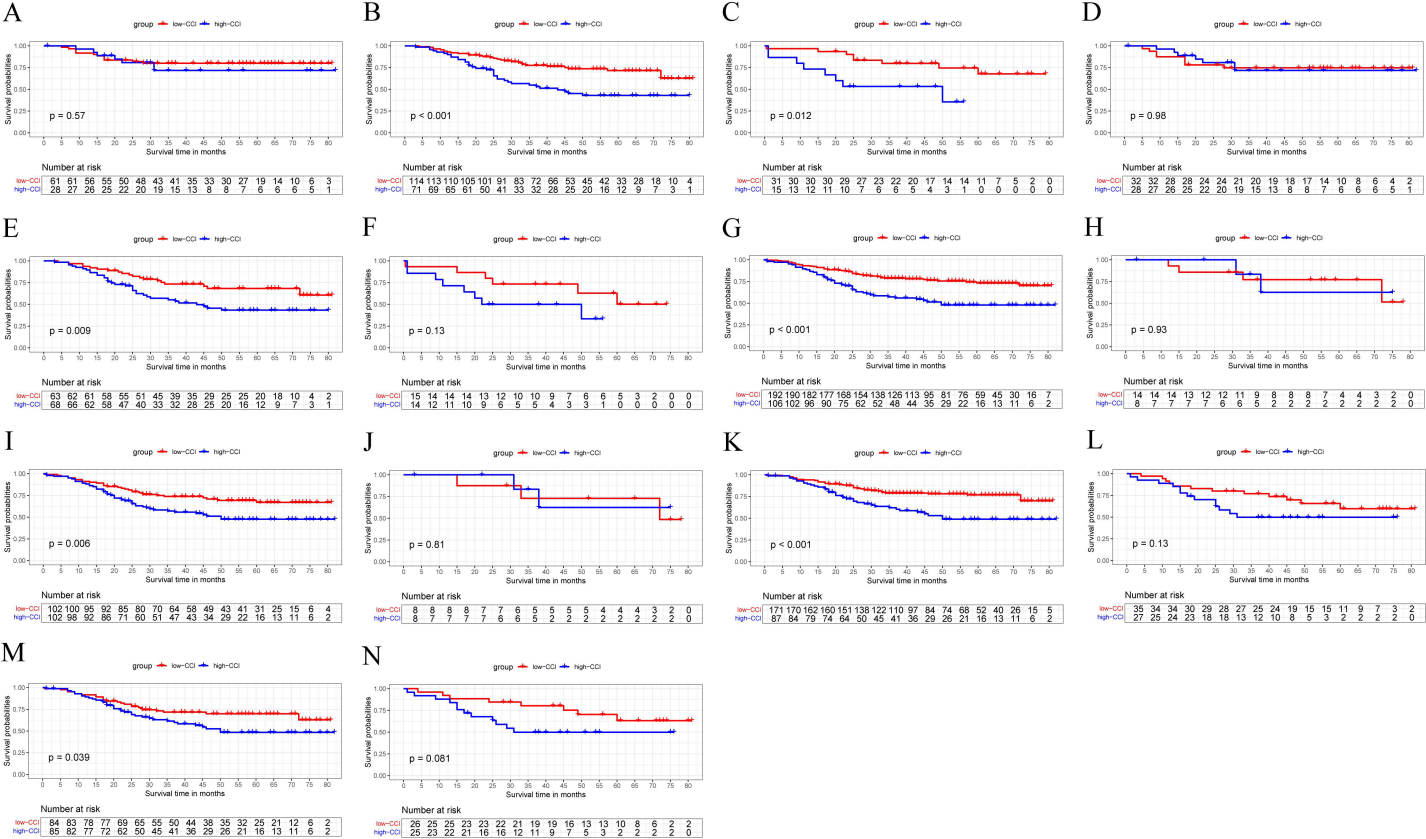
The Kaplan-Meier curve analysis between the high-CCI group and the low-CCI group in the subgroup of low-NPS **(A, D)**, medium-NPS **(B, E)**, high-NPS **(C, F)**, low-Charlson index **(G, I)**, high-Charlson index **(H, J)**, age ≤ 65 **(K, M)** and age > 65 **(L, N)** before and after PSM.

Before PSM, patients with a higher CCI exhibited significantly worse survival outcomes within the subgroups of medium and high Naples scores compared to those with a lower CCI (**Figures 3B, C**). However, there were no survival difference between two group in the subgroup of low Naples score (**Figure 3A**). Following PSM, the survival advantage of the low CCI group remained evident in the medium Naples subgroup (**Figure 3E**). Additionally, in the high Naples score subgroup, a trend toward improved survival was observed in low-CCI group (**Figure 3F**). Similarly, no survival difference was observed between two group in the subgroup of low Naples score after PSM (**Figure 3D**).

A statistically significant difference in survival outcomes between the high and low CCI groups was observed both before and after PSM in the subgroup of patients with a low charlson comorbidity score (**Figures 3G, I**). Similarly, a significant survival disparity between these two groups was also present in the subgroup of patients aged≤65 years, as is shown in **Figures 3K, M**. While, the low CCl group had similar survival with the high CCI group in terms of patients with high charlson score (**Figures 3H, J**)and patients aged over 65 (**Figures 3L, N**).

### 3.5 Univariate and multivariate logistic analysis of high CCI

By the receiver operating characteristic curve, the operative time was divided into long duration group (≥278.5mins) and short duration group(<278.5mins). Univariate logistics indicated that clinical T stage, clinical N stage, intraoperative blood loss, and operation time were potential risk factors for high CCI(P<0.05). Further multivariate logistic analysis using the forward method confirmed that intraoperative bleeding (P = 0.003) was independent risk factors of high CCI, as is shown in **Supplementary Table S4**.

## 4 Discussion

Early postoperative complications reflect short-term outcomes and may negatively affect the long-term prognosis by prolonging inflammation and increasing immunosuppression, thus promoting tumor recurrence and metastasis (24–26). Studies have shown that maintaining strong cellular immunity and controlling excessive catecholamine and prostaglandin responses during the perioperative period can reduce immune suppression, tumor recurrence, benefiting both short- and long-term survival (27). However, the link between postoperative complications and long-term survival in EC patients remains unclear (28–30). While complications generally harm long-term survival, the system of Clavien-Dindo grades have not shown significant prognostic differences in ESCC (31, 32). This may be due to the low sensitivity of Clavien-Dindo grading in predicting survival outcomes. Recently, the concept of textbook outcome has been introduced as a new way to assess complications and predict prognosis in ESCC (33). However, textbook outcome still relies on Clavien-Dindo grading, which may not fully capture the complexity of complications. Previous studies suggested that the CCI was more sensitive than traditional classifications (34–36). Therefore, this study aimed to evaluate the value of CCI in predicting both short- and long-term outcomes in ESCC patients.

Our results showed that the survival outcomes between higg-CCI group and low-CCI group were statistically different. The Kaplan-Meier survival analysis indicated that patients with high CCI had worse prognosis both before and after PSM. Cox analysis further confirmed that CCI was an independent risk factor of OS. This was consistent with the report from Kudo T et al. (37). However, therer are several advantages of our study. First, the larger sample size strengthened the reliability of our findings. Second, surgical approaches for esophageal cancer differ in their impact on postoperative complications; therefore, we focused on patients who underwent the McKeown procedure to minimize confounding. Third, subgroup analysis confirmed the prognostic value of CCI. Notably, this value was evident not only in patients with moderate to high Naples scores, but also in those with low Charlson index scores and in younger patients. This suggests that CCI-based assessment of complications provides independent prognostic information that complements existing risk stratification tools, even in traditionally low-risk groups.

Thoerically, CCI could provide a more comprehensive evaluation of postoperative complications, more accurately reflecting the severity of postoperative complications and the influence of postoperative complications on the level of systemic inflammatory response and degree of immunosuppression (38). For example, for patients experiencing one Clavien-Dindo grade 2 complication or combined with two grade 1 postoperative complications, the CCI score does not exceed 24.2. In contrast, for patients with at least one complication of Clavien-Dindo grade 3 or higher (severe complications), the CCI score exceeds 24.2. When the Clavien-Dindo classification system is used to evaluate the impact of postoperative complications on prognosis, the assessment typically considers only the most severe complication, thereby overlooking the contribution of less severe events. Therefore, utilizing the CCI for a comprehensive evaluation of postoperative complications allows for a more holistic understanding of their impact on long-term patient outcomes.

Compared with other complication grading methods, CCI is more strongly correlated with length of stay and cost of stay (39). This study supported that the high-CCI group had longer hospital stays and higher surgical costs. Longer hospital stays and higher medical costs were a huge economic and psychological burden for patients and their families, which suggested that we should actively prevent and effectively treat complications. Meanwhile, given the strong correlation between CCI and postoperative hospitalization costs, CCI may be a potential reference index for insurance settlement.

T stage and N stage were aslo identified as independent risk factors of OS, which is similar with other studies (40). In this study, the Naples score and Charlson status were not identified as independent risk factors. This outcome may be attributed to the fact that these two measures primarily reflect short-term nutritional status and, consequently, are insufficient to comprehensively capture long-term nutritional conditions (16). Current evidence suggests that the clinical benefits of adjuvant therapy may vary depending on the patient’s condition. Adjuvant therapy is associated with survival benefits in patients with lymph node positivity (41), but not in those without lymph node metastasis (42). Therefore, when developing individualized treatment strategies, it is important to evaluate the patient’s clinical status, the risk of postoperative complications, and the potential benefits of adjuvant therapy to support more precise and tailored decisions. Smoking status is not identified as an independent risk factor for OS, and this finding aligns with previous research (43). This study also found more intraoperative blood loss were associated with high CCI, which was similar with previous study (44).

This study has several limitations. Despite the establishment of stringent criteria, certain limitations inherent to retrospective matched analyses were unavoidable. Additionally, treatment approaches for similar postoperative complications may differ across centers or groups due to variations in clinical experience, potentially increasing heterogeneity among studies. Consequently, future research should aim to develop a standardized protocol for the evaluation and treatment of complications, based on multicenter studies, to enhance the assessment of prognostic impacts and facilitate cross-center comparisons. Moreover, due to the relatively small population of patients with esophageal adenocarcinoma (EAC), this study included only ESCC patients. As a result, the applicability of CCI in EAC patients requires further validation. Furthermore, this study excluded patients who underwent surgical treatment following neoadjuvant therapy, necessitating additional validation of the CCI scoring system’s applicability in this specific patient population. Future studies with larger sample sizes are essential to confirm our findings.

## 5 Conclusion

The CCI has the capability to quantify postoperative complications following esophagectomy. Before and after PSM, a high CCI is correlated with extended postoperative hospital stays and increased hospital costs. Furthermore, it serves as an independent risk factor for poor OS, holding significant potential as a reference tool for insurance claims and offers valuable evaluation indicators for clinicians concerning surgical quality control and patient prognosis management.

## Data Availability

The original contributions presented in the study are included in the article/**Supplementary Material**. Further inquiries can be directed to the corresponding authors.
