# Supplementary material for: High comprehensive complication index after minimally invasive esophagectomy associated with poor short-term and long-term outcome: a propensity score matching analysis
Source: Front Oncol. 2025 Oct 28;15:1661797. doi: 10.3389/fonc.2025.1661797 (PMC12602203; doi:10.3389/fonc.2025.1661797)
Supplement: Supplementary file 1 [file Table1.docx]

Table S1. The detail information of ESCC patients.

| Variables | N(%) |
| --- | --- |
|  |  |
| Sex |  |
| male | 240 (75.00%) |
| female | 80 (25.00%) |
| Age |  |
| ≤65 | 258 (80.62%) |
| ＞65 | 62 (19.38%) |
| BMI |  |
| ≤ 18.5 | 31 (9.69%) |
| 18.5–25 | 238 (74.38%) |
| ＞25 | 51 (15.94%) |
| Charson comorbidity index |  |
| 0-1 | 298 (93.12%) |
| ≥2 | 22 (6.88%) |
| Tumor location |  |
| upper | 29 (9.06%) |
| middle | 223 (69.69%) |
| lower | 68 (21.25%) |
| Smoking history |  |
| no | 133 (41.56%) |
| yes | 187 (58.44%) |
| CEA |  |
| normal | 290 (90.62%) |
| abnormal | 30 (9.38%) |
| Vascular tumor thrombus |  |
| negative | 247 (77.19%) |
| positive | 73 (22.81%) |
| T stage |  |
| T1 | 104 (32.50%) |
| T2 | 49 (15.31%) |
| T3 | 167 (52.19%) |
| N stage |  |
| N0 | 178 (55.62) |
| N1 | 76 (23.75) |
| N2 | 56 (17.5) |
| N3 | 10 (3.12) |
| Grade |  |
| G1 | 119 (37.19%) |
| G2 | 170 (53.12%) |
| G3 | 31 (9.69%) |
| NPS |  |
| 0 | 89 (27.81%) |
| 1-2 | 185 (57.81%) |
| 3-4 | 46 (14.38%) |
| Adjuvant therapy |  |
| 0 | 153 (47.81%) |
| 1 | 167 (52.19%) |
| lymphagectomy |  |
| 2-field | 302 (94.38%) |
| 3-field | 18 (5.62%) |

**Table S2** The comparisons of LOS and hospital cost between high CCI group and low CCI group before and after PSM.

|  | Before PSM | | | After PSM | | |
| --- | --- | --- | --- | --- | --- | --- |
|  | low CCI group | high CCI group | P | low CCI group | high CCI group | P |
| Medial cost  M (Q₁, Q₃) | 84832.46  (77603.92, 91091.50) | 97098.51  (86882.66, 122898.31) | <0.001 | 84590.86  (77811.55, 89662.26) | 96342.50  (86246.75, 122015.32) | <0.001 |
| LOS  M (Q₁, Q₃) | 9.00 (9.00, 11.00) | 16.00 (12.00, 25.00) | <0.001 | 9.00 (9.00, 11.75) | 16.00 (11.25, 24.75) | <0.001 |

**Table S3.** The univariate and multivariate logistic analysis of high CCI(>24.2) before matching.

| Variables |  |  |  |  |
| --- | --- | --- | --- | --- |
|  | OR (95%CI) | P | OR (95%CI) | P |
| Sex |  |  |  |  |
| male | Reference |  |  |  |
| female | 0.71 (0.41 ~ 1.23) | 0.226 |  |  |
| Age |  |  |  |  |
| ≤65 | Reference |  |  |  |
| ＞65 | 1.52 (0.86 ~ 2.67) | 0.148 |  |  |
| BMI |  |  |  |  |
| ≤ 18.5 | Reference |  |  |  |
| 18.5–25 | 2.12 (0.88 ~ 5.13) | 0.094 |  |  |
| ＞25 | 1.57 (0.56 ~ 4.39) | 0.392 |  |  |
| Charson comorbidity index |  |  |  |  |
| 0-1 | Reference |  |  |  |
| ≥2 | 1.04 (0.42 ~ 2.55) | 0.940 |  |  |
| Tumor location |  |  |  |  |
| upper | Reference |  |  |  |
| middle | 0.75 (0.34 ~ 1.64) | 0.469 |  |  |
| lower | 0.82 (0.34 ~ 2.00) | 0.669 |  |  |
| Smoking history |  |  |  |  |
| no | Reference |  |  |  |
| yes | 1.44 (0.90 ~ 2.30) | 0.131 |  |  |
| Intraoperative blood loss |  |  |  |  |
| ＜100ml | Reference |  | Reference |  |
| 100-200ml | 2.62 (1.52 ~ 4.51) | <0.001 | 2.62 (1.52 ~ 4.51) | <0.001 |
| ＞200ml | 2.97 (1.22 ~ 7.22) | 0.016 | 2.97 (1.22 ~ 7.22) | 0.016 |
| NPS score |  |  |  |  |
| 0 | Reference |  |  |  |
| 1-2 | 1.36 (0.79 ~ 2.32) | 0.265 |  |  |
| 3-4 | 1.05 (0.49 ~ 2.26) | 0.892 |  |  |
| Operation time |  |  |  |  |
| ＜278.5mins | Reference |  |  |  |
| ＞278.5mins | 1.65 (1.04 ~ 2.63) | 0.035 |  |  |
| Field |  |  |  |  |
| 2-filed | Reference |  |  |  |
| 3-field | 1.48 (0.57 ~ 3.86) | 0.424 |  |  |
